# Variants of *CARD8* in *Leishmania guyanensis*-cutaneous leishmaniasis and influence of the variants genotypes on circulating plasma cytokines IL-1β, TNFα and IL-8

**DOI:** 10.1371/journal.pntd.0011416

**Published:** 2023-06-05

**Authors:** Héctor David Graterol Sequera, Josué Lacerda de Souza, José do Espírito Santo Junior, Lener Santos da Silva, Suzana Kanawati Pinheiro, Herllon Karllos Athaydes Kerr, Mara Lúcia Gomes de Souza, Marcus Vinitius de Farias Guerra, Tirza Gabrielle Ramos de Mesquita, Rajendranath Ramasawmy

**Affiliations:** 1 Programa de Pós-Graduação em Medicina Tropical, Universidade do Estado do Amazonas, Manaus, Brazil; 2 Faculdade de Medicina Nilton Lins, Universidade Nilton Lins, Manaus, Brazil; 3 Programa de Pós-Graduação em Imunologia Básica e Aplicada, Universidade Federal do Amazonas, Manaus, Brazil; 4 Programa de Pós-Graduação em Biodiversidade e Biotecnologia da Amazonia Legal (Rede Bionorte), Universidade do Estado do Amazonas, Manaus, Brazil; 5 Fundação de Medicina Tropical Doutor Heitor Vieira Dourado, Manaus, Brazil; 6 Genomic Health Surveillance Network: Optimization of Assistance and Research in the State of Amazonas (REGESAM), Manaus, Brazil; Oswaldo Cruz Institute, BRAZIL

## Abstract

Nucleotide-binding oligomerization domain, leucine-rich repeat-containing protein family (NLR) are intracellular pathogen recognition receptors mediating innate immunity, releasing proinflammatory cytokines IL-1β and IL-18, and promoting pyroptotic cell death, upon sensing pathogenic or endogenous danger signals. In animal models, NLRP3 inflammasome has a dual role, pathogenic or protective in *Leishmania*-infection, depending on the *Leishmania* species and mice strain. Caspase recruitment containing domain 8 (CARD8) is a negative regulator of NLRP3 inflammasome and also an inhibitor of transcription factor NFĸB, a major transcription factor of proinflammatory cytokines. We investigated whether single nucleotide variants in *CARD8* may partially explain why only a proportion of individuals coming from the same area of endemicity of leishmaniasis develop cutaneous leishmaniasis caused by *Leishmania guyanensis*. We genotyped four single nucleotide variants of the *CARD8* gene by direct nucleotide sequencing in 1741 individuals from an endemic area of leishmaniasis, constituting 850 patients with CL and 891 healthy controls. The frequencies of the genotypes of the variants rs2288877 T>C, rs73944113 C>T, and rs2043211 A>T are similar among the patients with CL and HC, while the variant rs2288876 A>G) reveals an excess of the genotype AA among the patients with CL (44%) compared to 37% in the HC group. Allele A of the variant rs2288876 A>G) is associated with susceptibility to CL (OR = 1.2 [95%CI 1.03–1.4]; P = 0.01). Haplotype analysis showed that individuals harboring the haplotype CCAA have 280% odds of developing CL caused by *L*. *guyanensis* (OR = 3.8 [95% CI 2.0–7.7]; p = 0.00004). The variants rs2288877 T>C and rs2288876 A>G correlate with the plasma level of IL-8. Spearman correlation showed a significant positive correlation between the rs2288876 A>G allele A and the level of IL-8 (ρ = 0.22; p = 0.0002). *CARD8* may partially contribute to the development of CL caused by *L*. *guyanensis*.

## Author summary

Leishmaniasis is a neglected tropical disease caused by species of the parasite Leishmania, transmitted by the sand fly bite. The disease manifests in different forms: asymptomatic, cutaneous, and visceral. The immunological response of the infected individual is critical in the clinical manifestation of the infection by Leishmania. Till today, we do not understand why some infected people with the parasite Leishmania, living in the same endemic area, develop the disease and others do not. Our work is to study the DNA of infected people developing the disease Cutaneous Leishmaniasis and compare it to those remaining and living in the same area. The gene *CARD8* has a relevant role in the regulation of immunological response. In this work, we look for differences in a small piece of DNA fragment of the gene *CARD8* to establish if the single nucleotide DNA sequence variation occurs more frequently in people developing the disease than people who do not. We showed here that some individuals who develop the disease possess a small fragment of DNA called haplotype of the CARD8 gene that may contribute to some people to progress to disease development. However, this does not mean that all individuals bearing this haplotype will develop the disease when infected with Leishmania.

## Introduction

Leishmania infection can cause a group of diseases called leishmaniases, a vector-borne parasitic disease caused by the intracellular protozoan parasite, *Leishmania* (*L*.). Clinical manifestations of *Leishmania* infection range from asymptomatic, mild skin self-healing or non-healing ulcers (localized cutaneous leishmaniasis CL), diffused CL, and disseminated CL to severe mucocutaneous destruction (mucocutaneous leishmaniasis; MCL) or deadly visceral Leishmaniasis (VL) [1]. CL, diffused CL, disseminated CL, and MCL are termed American Tegumentary Leishmaniasis (ATL) in the American continent.

Leishmaniasis, endemic in 97 countries, is ranked as the ninth-largest infectious disease burden and affects nearly 12 million people in tropical and subtropical areas [2]. In 2018, 253,435 new CL cases were reported worldwide [2]. In Brazil, 16,813 new cases of ATL were reported in 2020 [3], while only in the state of Amazonas, 1,690 new cases of ATL were notified [3]. More than 20 species of Leishmania cause leishmaniasis, a group of diseases. The common species that cause CL in Brazil are *L*. *braziliensis*, *L*. *guyanensis*, *L*. *lainsoni*, *L*. *naiffi*, and *L*. *lindbergi*, while *L*. *infantum/chagasi* is the etiological agent of VL. *L*. *guyanensis* is responsible for nearly 95% of CL in the state of Amazonas [4].

The outcome of *Leishmania* infection depends mainly on the species and the host’s immunological response and genetic background. Individual adaptive immune T helper cell responses and their interactions with parasitized host cells play pivotal roles in the resolution or evolution of the disease [5]. An early Th1 response during infection impedes the multiplication of the parasite by releasing sufficient levels of IFN-γ and superoxide production to protect the host from progressing to disease. By contrast, a Th2 response with high levels of interleukins-4, -5, and -13 favors parasite multiplication and disease progression [5,6].

The host immune system contains germ-line encoded pathogen-recognition receptors (PRRs), present on cell surfaces or intracellularly, to detect pathogens-associated molecular patterns (PAMPs). A group of intracellular PRRs, characterized by the presence of a caspase-recruitment domain (CARD) or pyrin domain (PYD), cooligomerizes with pro-caspase-1 (CASP1) to form high molecular weight inflammasomes complexes upon sensing their cognate ligands [7]. Inflammasome assembly in response to cytoplasm microbial PAMPs or danger signals (DAMPs) triggers CASP1 activation, leading to the secretion of IL-1β and IL-18, and also induces pyroptosis, an inflammatory form of programmed cell death via gasdermin D (GSDMD) [7].

NLRP3 inflammasome functions in tandem with a priming signal that leads to the activation of nuclear factor kappa B (NF-kB) and extracellular signal-related kinase (ERK) signaling pathways culminating in the transcription of NLRP3, pro-IL-1β, and pro-IL-18. Upon recognizing the appropriate DAMPs or PAMPs, occurs the activation and oligomerization of NLRP3. Oligomerization of NLRP3 triggers the association with apoptosis-associated speck-like protein containing CARD (ASC) and pro-caspase-1 via Pyrin-Pyrin and CARD-CARD homotypic interactions, respectively. Pro-caspase 1 converts to CASP1 to process pro-IL-1β and pro-IL-18 into active secreted forms of IL-1β and IL-18 [8,9]. The activation of the canonical NLRP3 inflammasome during *Leishmania* infection seemed to be through the Dectin-1/SYK/ROS signaling pathway, while the noncanonical is via the lipophosphoglycan-caspase-11 [10].

NLRP3 plays a relevant role in inflammation, releasing IL-1β and IL-18. Knockout mice for NLRP3, ASC, or CASP1 infected with *L*. *amazonensis* showed increased lesion size and parasite burden [11]. In vitro studies showed that macrophages infected with *L*. *braziliensis*, *L*. *amazonensis*, or *L*. *mexicana* required the NLRP3 inflammasome to release IL-1β [11,12]. Double-stranded *Leishmania* RNA virus (LRV) present in *L*. *guyanensis* inhibits caspase-1 and IL-1β production by activating TLR3 [13]. Bone marrow-derived macrophages (BMDM) infected with LRV^+^*L*. *guyanensis* displayed decreased caspase-1 and IL-1β levels than LRV- *L*. *guyanensis*-infected BMDM in vitro. C57BL6 mice infected with LRV^+^*L*. *guyanensis* showed large ear-swelling and high parasite load in comparison to LRV^-^*L*. *guyanensis* ear-infected C57BL6 mice. This difference decreases in NLRP3 knockout C57BL6 mice, suggesting NLRP3 and IL-1β protect against *L*. *guyanensis*-infection (de Carvalho RVH, Lima-Junior DS, 2019) [13].

Caspase recruitment containing domain family, member 8 (CARD8), also known as tumor-upregulated CARD-containing antagonist of caspase nine (TUCAN) or CARD inhibitor of NF-κB-activating ligands (Cardinal), also is a component of the NLRP3 inflammasome. CARD8 is a negative regulator of NLRP3 inflammasome [14]. CARD8 bears a FIIND domain that interacts with the NOD domain (FIIND-NOD) of NLRP3 [15] and a C-terminal CARD domain that interacts with CASP1 via the CARD-CARD domain through homophilic interactions [16]. Blocking *CARD8* expression by small interfering RNA increases the production of IL-1β [14]. CARD8 also inhibits NFkB activity to decrease the expression of IL-1 β and TNFα [17,18].

Inhibition of the NLRP3 inflammasome in cell cultures prepared from skin lesion biopsies of patients with CL caused by *L*. *braziliensis* exhibit low release of IL-1β [19]. Caspase 1/11 and NLRP3 knockout mice infected with *L*. *braziliensis* manifest mild pathology than wild-type mice [19]. A transcriptional study of skin biopsy specimens of ulcer lesions from *L*. *braziliensis*-infected patients with CL showed an upregulation of IL1B and genes involved in the NLRP3 inflammasome [20]. In patients with CL caused by *L*. *mexicana*, level of IL-1β is proportional to disease severity [12].

Together, these studies in animal models of leishmaniasis suggest that any component of the NLRP3 inflammasome may have a role in the pathogenesis of leishmaniasis. Notably, NLRP3 inflammasome releases CASP1 to process pro- IL-1β and pro-IL-18 to active IL-1β and IL-18. CARD8 acts as a negative regulator of the NLRP3 inflammasome and may control the processing of pro-IL-1 β by inhibiting CASP1. In light of the importance of IL-1 β in the pathology of CL or protection against *Leishmania* infection, genetic variants in the *CARD8* gene may be associated with susceptibility or resistance to leishmaniasis. Indeed, genetic variants in the *CARD8* gene are associated with several inflammatory diseases [21]. In this study, we hypothesized that genetic variants in the *CARD8* gene might partially explain why some individuals progress to the development of CL while others do not despite living in the same area of endemicity of leishmaniasis caused principally by *L*. *guyanensis*.

## Materials and methods

### Ethical statements

This study was conducted according to the principles expressed in the Declaration of Helsinki and was approved by the Research Ethics Committee of the Fundação de Medicina Tropical Dr. Heitor Vieira Dourado granted under the file number CAAE:09995212.0.0000.0005. All the participants provided written informed consent for biological sample collection and subsequent analysis. For participants less than 18 years of age, the parent/guardian provided written informed consent for biological sample collection and subsequent analysis.

### Area of the study population

The study included 1741 unrelated individuals from the same endemic region of CL caused by *L*. *guyanensis* and are previously described [22–24]. The study was performed at the Fundação de Medicina Tropical Dr. Heitor Vieira Dourado (FMT-HVD), the reference center for tropical diseases. All of the participants were from the perirural area of Manaus, the capital city of the Amazonas state, Brazil. Briefly, all the participants are from the areas surrounding BR-174 and AM-010 that became an area of endemicity for *L*. *guyanensis-* infection due to human invasion (i.e., communities of Pau-Rosa, Cooperativa, Água-Branca, Leão, and Brasileirinho). The population is an admixture of Native American (50 to 60%), European (40 to 50%), and African (around 10%) ancestries [25].

### Sample size calculations

Sample size calculation for performing a case-control study on the immunogenetics of *Lg*-Cl was previously described [26]. Briefly, the effective sample size was calculated rigorously for a case-control study (based on a trait with inputs of multiple genes) online using the Genetic Power calculator of Harvard University (http://pngu.mgh.harvard.edu/~purcell/gpc) under the assumptions of a minor allele frequency of 5%, disease prevalence of 5%, complete linkage disequilibrium 1 between marker and trait, case-control ratio 1 and 5% type1 error rates with an odds ratio of 1.5 and 2.0 for heterozygosity and homozygosity, respectively. For 80% power, the genetic allelic model generated a sample size of 789 for cases and 789 for controls.

### Collection of Biological Samples and DNA isolation

Five mL of peripheral blood from each participant was drawn by venipuncture and collected into EDTA-containing Vacutainers (Becton Dickinson, Sao Paulo, Brazil) for genomic DNA isolation by the proteinase K salting-out method [27] and measurement of plasma circulating cytokines.

### Identification of Leishmania species

Before proceeding with a biopsy specimen collection of the skin ulcer lesion from all the patients with CL to identify the *Leishmania* sp., confirmation of the presence of the parasite was by direct microscope examination of Giemsa-stained in the lesion scarifications. DNA from the biopsy specimens of all the patients with CL underwent *Leishmania vianna*-specific PCR with discrimination between *L*. *braziliensis* and *L*. *guyanensis* according to protocols previously described [28,29]. Species identification was also realized by direct nucleotide sequencing of a fragment of the HSP70 (233bp) and mini exon genes (227bp) [30] with the kit BigDye Terminator v3.1 Cycle Sequencing (Thermofisher, MA USA) following the protocols suggested by the manufacturer.

### Genotyping of *CARD8* variants

We designed a pair of primers (CARD8F 5`-GGTTCTCTAGACACCTCCATGC-3’ and CARD8R 5’-GCACAGCCTATGCTATCATCAGG-3’) flanking four SNVs (rs2288877 T>C; rs73944113 C>T; rs2043211 A>T; rs2288876 A>G) located in the intron 5 and exon 6 of the CARD8 gene. DNA extracted from whole blood was amplified with the pair of primers to generate a fragment of 317 bp by polymerase chain reaction. PCR reactions were performed in the Applied Biosystem Veriti Thermal Cycler and consisted of 50 ng of genomic DNA in a final volume of 25 μL containing 1.5 mmol/L of MgCl2, 0.25 pmol/L of forward and reverse primers, 40 μmol/L of each dNTP (dideoxynucleotide triphosphate) and 1 U of Taq polymerase in buffer containing 100 mmol/L of Tris-HCL (pH 8.3) and 500 mmol/L KCL. The PCR cycling conditions were an initial denaturation step of 5 minutes at 95°C followed by 35 cycles of 15 s at 95°C, 15 s at 60°C, 30 s at 72°C and a final extension step at 72°C for 7 minutes. PCR products were purified with 20% PEG following the protocols described elsewhere [31,32]. The amplicons 317bp were sequenced with either the forward (CARD8F) or reverse (CARD8R) primers using the Kit BigDye Terminator v3.1 Cycle Sequencing (Thermofisher, MA USA) according to the manufacturer’s protocol in the Applied Biosystem Veriti Thermal Cycler. The sequencing product was run in an ABI 3130XL automatic DNA sequencer, using POP-7. Reading of nucleotides was done by the use of the Sequencing Analysis software (Applied Biosystems, v5.3.1), and only high-quality sequences were used for SNP analyses.

### Assay of IL-1β, TNFα and IL-8 in plasma

The assay of circulating plasma levels of IL-1β, TNFα, and IL-8 was done with the Bio-Plex Pro-Human Cytokine Grp I Panel 27-plex Kit (Bio-Rad, Hercules, CA USA), following the manufacturer’s instructions in the Bioplex 200 Protein Array System (Luminex Corporation, Austin, TX, USA).

### Statistical analyses

Statistical analysis, for comparison of genotypes and alleles between patients with CL and HC, was performed using logistic regression analysis from the website https://ihg.helmholtz-muenchen.de/ihg/snps.html that also calculated deviation from Hardy–Weinberg expectation. Linkage disequilibrium, as well as comparisons of haplotypes, was performed with the Haploview software 4.2. Inheritance models (codominant, dominant, recessive, and overdominant models) were analyzed by the R software version 4.0.0 of the SNPassoc package. Effects of genotypes on circulating plasma cytokine levels of IL-1β, TNFα and IL-8 were realized with the R software version 4.0.0 of the SNPassoc package for quantitative traits using the Generalized Linear Model and ggplot2 package for visualizing.

## Results

### The study population

Of the 1741 individuals included in the study, 850 had CL and the remaining 891 individuals with no history and scar of leishmaniasis are considered healthy controls (HC). The healthy individuals are from the same endemic area of the patients with *L*. *guyanensis*-CL (*Lg*-CL) and were physically examined by physicians to exclude any doubts of history of leishmaniasis. They share the same socio-epidemiological environment and have been living in the endemic area for more than five years. The majority of the participants in this study are agricultural or farm workers. All the participants are unrelated individuals. This case-control study compares unrelated patients with *Lg*-CL to healthy unrelated individuals. The study followed the guidelines strengthening the reporting of genetic association studies (STREGA). All the patients had active CL and fewer or equal to six ulcer skin lesions. Of the patients with *Lg*-CL, 639 are male with a mean age of (mean ± standard deviation) 34.4±13.7 years old, while the remaining 211 female participants had a mean age of 37.5±15.7 years. In the HC group, there were 608 male (42±17.5 years) and 283 female (40±17.4 years) participants. All the participants were devoid of HIV, cardiac, renal, or diabetic diseases. HC male participants were slightly older than male patients with *Lg*-CL (p< 0.0001).

### Genotypes and allele frequencies of the *CARD8* variants

**Fig 1:**
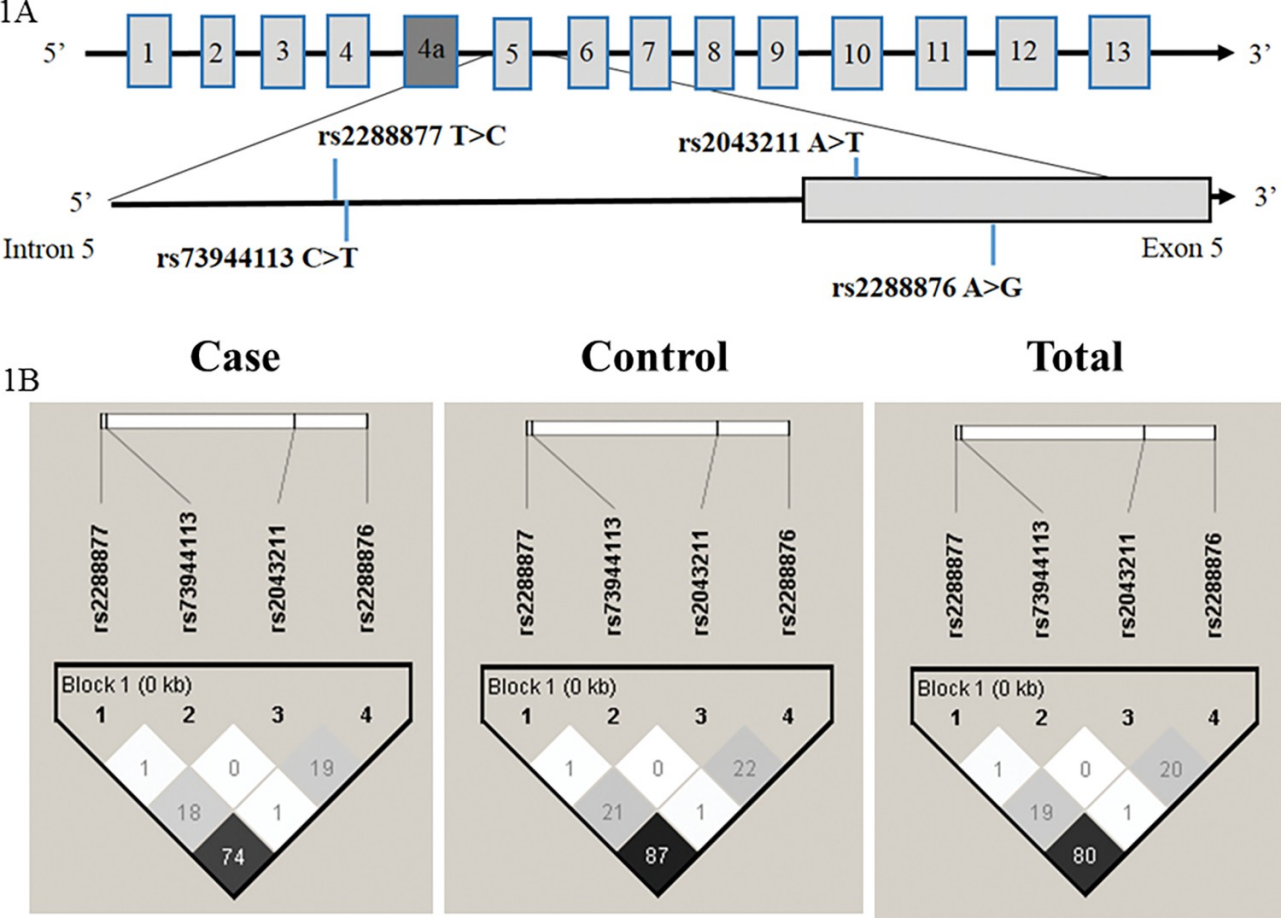
Localization of the four variants on the *CARD8* gene is depicted in 1A and linkage disequilibrium between the variants among patients with cutaneous leishmaniasis (cases), healthy controls, and all participants of the study (Totals) in 1B.

*CARD8* is located on the long arm of chromosome 19 and possesses 13 exons. The variants rs2043211 A>T and rs2288876 A>G are located in exon 5 while rs2288877 T>C and rs73944113C>T are in intron 5. The variant rs2043211 A>T is a transversion of Adenine to Thymine, introducing a stop codon at codon ten (Cys10stop), while the rs2288876 A>G is a transition of Adenine to Guanine, creating a nonsense mutation. The localization of the four SNVs on the *CARD8* gene and linkage disequilibrium (LD) between the four SNVs are shown in Fig 1A and 1B, respectively.

The genotypes distributions of the four *CARD8* SNVs (rs2288877 T>C; rs73944113 C>T; rs2043211 A>T; rs2288876 A>G) are under the Hardy-Weinberg Equilibrium expectation in both groups of patients with CL and HCs group. Genotyping success rate was 95% for rs2288876 A>G and rs2043211 A>T, while 94% for rs2288877 T>C and rs73944113 among the patients with *Lg*-CL. Within the HC, genotyping success rate was 89% for rs2288876 A>G and rs2043211 A>T and 88% for rs2288877 T>C and rs73944113.

Table 1 shows the frequencies of the genotypes and alleles of the four SNVs. Of the four SNVs, only the variant rs2288876 A>G showed a different distribution of genotypes between the patients with CL and HC. The frequencies of the genotypes of the variants rs2288877 T>C and rs2043211 A>T are similar among the patients with CL and HC, while the variant rs2288876 A>G) showed an excess of homozygotes AA among the patients with CL (44%) compared to 37% in the HC group. Similarly, the variant rs73944113 C>T showed an excess of heterozygotes among the patients (41/799; 5%) compared to 3.4% (27/783) in the HC group.

**Table 1:** Genotypes and alleles frequencies of the variants rs2288877 T>C, rs73944113 C>T, rs2043211 A>T, and rs2288876 A>G among the patients with *L*. *guyanensis*-cutaneous leishmaniasis (cases) and healthy controls (controls).

	Genotypic and allelic frequencies			
Genotype/ allele	Cases	%	Controls	%
**rs2288877 T>C**	*N = 796*		*N = 782*	
T/T	341	42.83	306	39.13
T/C	356	44.72	373	47.69
C/C	99	12.45	103	13.18
T	1038	65.20	985	62.97
C	554	34.80	579	37.02
**rs73944113 C>T**	*N = 799*		*N = 783*	
C/C	757	94.74	755	96.42
C/T	41	5.13	27	3.44
T/T	1	0.13	1	0.14
C	1555	97.30	1537	98.14
T	43	2.69	29	1.85
**rs2043211 A>T**	*N = 806*		*N = 793*	
A/A	396	49.13	414	52.20
A/T	337	41.81	321	40.47
T/T	73	9.06	58	7.33
A	1129	70.03	1149	72.44
T	483	29.96	437	27.56
**rs2288876 A>G**	*N* = 809		*N = 793*	
A/A	354	44	292	37
A/G	357	44	395	50
G/G	98	12	106	13
A	1065	65.82	979	61.72
G	553	34.17	607	38.27

Table 2 shows the statistical comparisons of the genotypes’ frequencies between the patients with *Lg*-CL and HC. There are no significant differences for genotypes and allele frequencies of rs2288877 T>C; rs73944113 C>T; rs2043211 A>T between patients with *Lg*-CL and HC. Homozygous AA individuals for the variant rs2288876 A>G have 30% higher odds of developing CL (OR = 1.3 [95% CI 1.1–1.7]; P = 0.006) than heterozygous individuals. The allele A is associated with susceptibility to CL (OR = 1.2 [95%CI 1.03–1.4]; P = 0.01).

**Table 2:** Statistical comparisons of the genotypes and alleles frequencies of the single nucleotide variants (rs2288877 T>C; rs73944113 C>T; rs2043211 A>T; rs2288876 A>G) using linear regression analysis (https://ihg.helmholtz-muenchen.de/ihg/snps.html) between patients with cutaneous leishmaniasis caused *L*. *guyanensis* (cases) and healthy controls coming from the same endemic areas as patients (Controls).

	Comparisons	OR [95% CI]	OR [95% CI] Reciprocal	*p-*value
**rs2288877 T>C**	T/T vs C/C	0.86 [0.63–1.20]	1.2 [0.83–1.6]	0.35889
	T/T vs T/C	0.86 [0.69–1.06]	1.2 [0.94–1.4]	0.15174
	T/T vs C/C+ T/C	0.86 [0.70–1.05]	1.2 [0.95–1.4]	0.13422
	T vs C	0.91 [0.79–1.05]	1.1 [0.95–1.3]	0.19335
**rs73944113 C>T**	C/C vs T/T	1.0 [0.06–6.0]	1.0 [0.17–17]	0.99851
	C/C vs T/C	1.5 [0.92–2.5]	0.67 [0.4–1.08]	0.09890
	C/C vs T/T+ T/C	1.5 [0.92–2.4]	0.67 [0.42–1.08]	0.10413
	C vs T	1.5 [0.91–2.4]	0.67 [0.42–1.08]	0.11358
**rs2043211 A>T**	A/A vs T/T	1.3[0.91–1.9]	0.77 [0.53–1.08]	0.14653
	A/A vs A/T	1.1 [0.89–1.3]	0.91 [0.77–1.1]	0.37521
	A/A vs T/T+A/T	1.1[0.93–1.4]	0.91 [0.71–1.1]	0.21877
	A vs T	1.1 [0.97–1.3]	0.91 [0.77–1.1]	0.13238
**rs2288876 A>G**	A/A vs G/G	0.76 [0.56–1.04]	1.3 [0.96–1.8]	0.09165
	A/A vs A/G	0.75 [0.60–0.92]	1.3 [1.1–1.7]	0.00631
	A/A vs G/G+A/G	0.75 [0.61–0.92]	1.3 [1.1–1.7]	0.00467
	A vs G	0.84 [0.72–0.97]	1.2 [1.03–1.4]	0.01591

**Abbreviations:** OR = Odds Ratio; CI = Confidence interval; *p*-value <0.05 significant.

Table 3 shows the inheritance models of variant rs2288876 of *CARD8* as performed by the R statistics package. According to the Akaike Information Criteria, the best model is the dominant inheritance showing homozygous individuals for the A allele have 36% odds of developing CL if infected by *L*. *guyanensis* (p-value and OR adjusted for age and sex Pv = 0.003; [OR = 1.36 [95% CI 1.10–1.67]) compared to individuals bearing the G allele. Conversely, carriers of the G allele have a 26% (OR = 1/1.36 = 0.74) fewer odds of developing *Lg*-CL.

**Table 3:** Inheritance models of the variant rs2288876 of *CARD8*.

rs2288876		*p-*value [OR (95% CI)]	*Adj p*-value [OR (95% CI)]
Dominant	A/A vs G/A+G/G	0.004 [1.33 (1.09–1.63)]	0.003 [1.36 (1.10–1.67)]
Recessive	A/A+G/A vs G/G	0.451 [1.12 (0.83–1.50)]	0.37 [1.15 (0.85–1.55)]
Over dominant	A/A+G/G vs G/A	0.015 [1.26 (1.03–1.53)]	0.02 [1.26 (1.03–1.55)]

**Abbreviations:** *p-*value [OR (95% CI)] = *p*-value <0.05 significant and Odds Ratio unadjusted for age and sex; *Adj p-*value [OR (95% CI)] = *p*-value <0.05 significant and Odds Ratio adjusted for age and sex.

Construction of haplotypes was via the Haploview software v.4.2. Complete genotyping data for the four SNVs among patients with CL and HC are 790 and 776, respectively. Table 4 shows the six haplotypes observed in the study population.

**Table 4:** Distribution of haplotypes constructed with the single nucleotide variants (rs2288877 T>C; rs73944113 C>T; rs2043211 A>T; rs2288876 A>G) of *CARD8* among patients with cutaneous leishmaniasis caused *L*. *guyanensis* (Cases) and healthy controls coming from the same endemic areas as patients (HC).

Haps	SNV 1	SNV 2	SNV 3	SNV 4	Cases n (%)	HC n (%)	χ^2^	OR [95% CI]	*p-*value
**1**	C	C	A	G	492 (32)	557 (36)	7.93	0.8[0.7–0.9]	0.004
**2**	T	C	A	A	498 (32)	500 (32)	0.17	1.0[0.9–1.2]	0.67
**3**	T	C	T	A	450 (29)	415 (27)	1.10	0.91[0.78–1.0]	0.29
**4**	T	T	A	A	41 (2.4)	29 (1.8)	1.89	0.71[0.44–1.1]	0.16
**5**	T	C	A	G	34 (2.0)	30 (1.8)	0.31	0.86[0.51–1.4]	0.57
**6**	C	C	A	A	42 (2.6)	12 (0.8)	17	3.8[2.0–7.7]	3.7E-5

**Abbreviations:** Haps, Haplotypes; SNV, Single nucleotide variant; 1, rs2288877 T>C; 2, rs73944113 C>T; 3, rs2043211 A>T and 4 rs2288876 A>G according to the position in the *CARD8* gene; **χ**^**2**^, Chi-square test; OR, odds ratio; CI, confidence Interval; *p*-value <0.05 significant.

The SNV rs2288877 T>C and rs2288876 A>G showed strong LD in both patients and HC groups, as shown in Fig 1B. Carriers of haplotype 1 (CCAG) have 20% lower odds of developing CL (OR = 0.8 [95% CI 0.7–0.9]; p = 0.004), while the carrier of haplotype 6 (CCAA) bears 280% odds of developing CL caused by *L*. *guyanensis* (OR = 3.8 [95% CI 2.0–7.7]; p = 0.00004).

### Comparison of circulating plasma levels of IL-1β, TNFα and IL-8 with the SNVs genotypes

CARD8 inhibits the transcription factor NFĸB and leads to diminishing expression of IL-1β and TNFα [17,18]. As CARD8 protein lacking the first ten amino acids due to the variant rs2043211 A>T, was shown to be unable to inhibit the transcription factor NFĸB, and homozygous T allele was correlated with high levels of IL-8 in whole blood stimulated with LPS [33], we investigated whether the genotypes of the different variants influence the plasma levels of IL-1β, TNFα, and IL-8. NLRP3 inflammasome release CASP1 to process pro-IL-1β and pro-IL-18 into active IL-1β and IL-18. Unfortunately, our Kit for cytokine assay did not include IL-18. We have previously shown that the plasma levels of IL-1β, TNFα, and IL-8 were higher among the patients with CL than HC [34]. Analysis of variance between plasma circulating levels of IL-1β, TNFα and IL-8 and the genotypes of either the rs73944113 C>T or rs2043211A>T did not reveal any significant correlations shown in S1 and S2 Figs, respectively.

**Fig 2:**
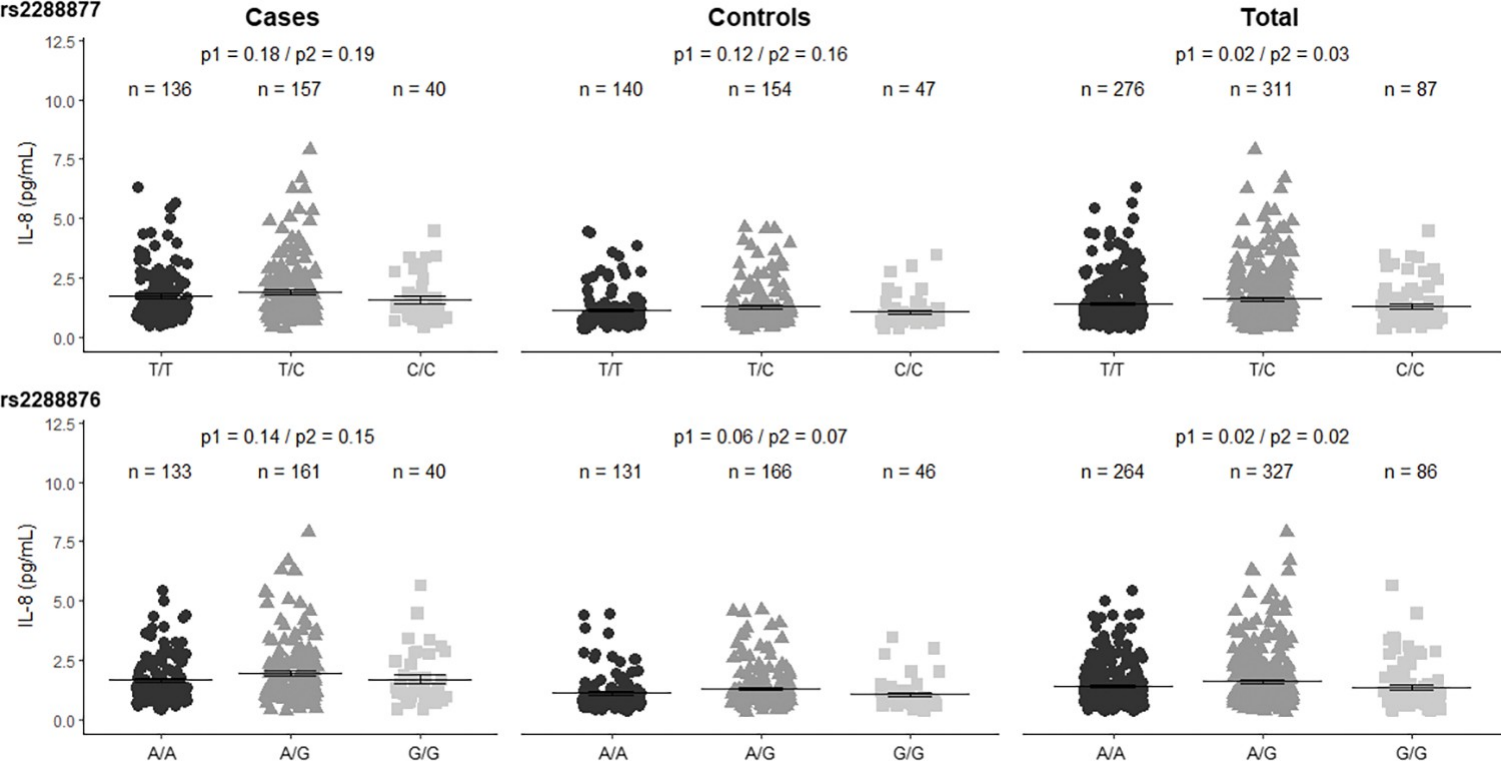
Correlation of circulating plasma IL-8 with the SNV rs2288877 T>C and rs2288876 genotypes of *CARD8* in the codominant model. Analysis was performed by the Generalized Linear Model using the Anova one-way parametric model. P1 is the *p*-value adjusted for age and sex, while P2 is the *p*-value unadjusted. The bar represents the mean expressed in picogram/mL while the error bar is the standard error of the mean.

Variance analysis between genotypes of rs2288877 and rs2288876 and plasma circulating levels of IL-1β, TNFα, and IL-8 showed a significant correlation with only IL-8 S3 and S4 Figs, respectively. Correlations of levels of IL-8 with rs2288877 and rs2288876 according to the codominant models are shown in Fig 2.

Homozygous individuals for the allele C of the rs2288877 seem to have lower levels of IL-8 (mean ± standard error of the mean: 1.3±0.09 pg/mL) than heterozygous individuals (1.6±0.07 pg/mL) and homozygous individuals (1.4±0.06 pg/mL) for the allele T among the total participants (patients with *Lg*-Cl + Healthy controls). The codominant model revealed a significant correlation between the genotypes with IL-8 (P adjusted for sex and age = 0.03) among the totals. In the overdominant genetic model, the CT genotype showed significant association by unpaired parametric T-test with the plasma level of IL-8 among the total participants (P adjusted for sex and age = 0.01), as shown S5 Fig.

Analysis of variance showed that the codominant model had significant correlations of IL-8 to the rs2288876 genotypes (P adjusted for sex and age = 0.02) among the total participant (patients with *Lg*-Cl + controls), and homozygous individuals for the G allele seem to have lower levels of IL-8. Heterozygous individuals had higher levels of IL-8 (1.62±0.07pg/mL) compared to homozygous individuals for the A allele (1.4±0.06 pg/mL) and to homozygous individuals for the G allele (1.4±0.10 pg/mL). Unpaired parametric T-tests revealed that the overdominant model best explained the correlation between the plasma levels of IL-8, as shown in S6 Fig. However, the Spearman correlation showed a significant positive correlation between the allele A and the level of IL-8 (ρ = 0.22; p = 0.0002), suggesting that the A allele may be the driving force influencing the level of IL-8 in plasma.

## Discussion

Identifying immune genes involved in the immunopathogenesis of *Lg* -CL may elucidate the immuno-mechanism contributing to the development of lesions in susceptible individuals and open the way for immunotherapy. We investigated four SNVs of *CARD8* and found that the allele A of the rs2288876 A>G and one of six haplotypes generated with the four SNVs were associated with susceptibility to the development of CL. The genotypes of rs2288876 A>G and rs2288877 T>C showed correlations with plasma circulating levels of IL-8.

TLR2, TLR4, and TLR9 recognize lipophosphoglycan, glycosphingophospholipids, and DNA of *Leishmania*, respectively. TLRs induce signaling in response to Leishmania infection to promote the production of inflammatory cytokines TNFα, IL-6, IL-1β, and IL-12 and may act as a priming step for the assembly of NLRP3 inflammasome through the transcription of NLRP3 via NFκB transcription factor [35]. Activation of NLRP3 inflammasome must be finely regulated to mount an appropriate immune response to provide protection and avoid damage to the host.

While several studies suggest a protective role of NLRP3 inflammasome in *Leishmania* infection [11,13,36–38], others propose a pathogenic role [19,39,40] BMDM from BALBc, primed with LPS and infected with *L*. *major*, secrete high levels of IL-1β and IL-18 while BMDM derived from Nlrp3^−/−^, Asc^−/−^, and Casp1^−/−^ knockout BALBc mice do not produce IL-1β and IL-18, suggesting NLRP3 inflammasome is necessary for the release of IL-1β and IL-18 [40]. However, clearance of intracellular *L*. *major* does not depend on NLRP3 inflammasome as both were able to eliminate the parasite [40]. Footpad infected with *L*. *major* showed increased swelling compared to NLRP3 deficient BALBc mice. NLRP3 inflammasome deficient mice showed higher IFNγ and lower IL-4 and IL-5, and the authors demonstrated that IL-18 drives a TH2 response and concluded that NLRP3 inflammasome might play a pathogenic role [40]. C57BL/6 wild-type mice ears infected with a non-healing strain of L. major exhibited high levels of IL-1β and IL-18 and chronic lesions compared to Nlrp3^−/−^, Asc^−/−^, Casp1^−/−^, and Il1b^−/−^ and Il1r^−/−^ mice that showed reduced parasite burden and healing lesion. The wild-type mice showed high numbers of neutrophils at the site of infection in the ear, that may be due to high IL-1β compared to deficient NLRP3 inflammasome mice [39]. Inhibition of NLRP3 inflammasome in mice infected with *L*. *braziliensis* confers resistance and protects the mice from developing lesions [20].

In light of these studies, NLRP3 can be protective or pathogenic, depending on the genetic background of the mice and the *Leishmania* sp. Interestingly, only a proportion of *Leishmania*-infected individuals in the area of endemicity for leishmaniasis progress to the development of the disease, suggesting genetic factors of the host may play a key role in either protecting or prone susceptibility to disease development besides other factors. The variant rs2043211 A>T of the *CARD8* gene has been associated with many inflammatory diseases, such as Crohn’s disease, rheumatoid arthritis, cardiovascular diseases, and ankylosing spondylitis [41–43]. The AA genotype of this variant was correlated with high NFĸB and IL-8, while the TT genotype with low NFĸB and IL-8 expression, suggesting CARD8 lacking the first ten amino acids loses the ability to inhibit NFĸB [33]. Of note, we refer to the A allele as the T allele based on the DNA strand transcribed, which means the nucleotide T will be A in the mRNA and introduce a stop codon by converting the codon TGT for cysteine to the stop codon TGA. The substitution of nucleotide A with T introduces a stop codon in the position of amino acid cysteine at codon 10 (Cys10X). The variant rs2043211 A>T is a transversion of adenine (A) to thymine (T). We did not observe any association of this variant with the development of CL. Variance analysis between the genotypes of rs2043211 A>T and plasma circulating IL-1β, TNFα and IL-8 revealed no significant correlations. Another study also did not reveal any variance of the rs2043211 A>T genotypes with serum levels of IL-1β among Crohn’s patients [44]. A study carried out with 1006 healthy blood donors also observed no association of rs2043211 A>T genotype with levels of IL-1β [45] while in another study in patients with arteriosclerosis, the TT genotype was associated with increased levels of IL-1β [46].

Referring to the SNV rs73944113 C>T, no association was revealed despite an excess of heterozygotes observed among the cases. Analysis of the variance of plasma IL-1β, TNFα, and IL-8 with the genotypes did not show any significant correlation. However, the rs2288876 A>G showed an association with the development of CL in susceptible individuals infected with *L*. *guyanensis*. This variant also correlated with plasma IL-8. The susceptible allele A seems to correlate with levels of IL-8. Similarly, the SNV rs2288877 T>C revealed a significant correlation with IL-8. Notably, this variant is in strong linkage disequilibrium with the SNV rs2288876 A>G and might reflect this correlation, but did not have any association with either susceptibility or resistance to CL development. However, the variants rs2288877 T>C and rs2288876 A> were associated with Crohn’s disease, an inflammatory disease, with an odds ratio of (OR = 1.3 [95% CI 1.1–1.67]; P = 0.04) and (OR = 1.28 [95% CI 0.96–1.69]; P = 0.07), respectively [47].

Haplotype analysis revealed that individual carrier of haplotype 6 (CCAA) has 280% odds of developing CL caused by *L*. *guyanensis* (OR = 3.8 [95% CI 2.0–7.7]; p = 0.00004), while haplotype 1 (CCAG) provides protection. Interestingly, the allele A of rs2288876 is distributed in several haplotypes as shown in Table 3, while the allele G is in only the protecting haplotype. Of note, the allele A correlated with a higher level of plasma IL-8, a potent chemoattractant of neutrophils. It can be assumed that only a subset of individuals bearing the A allele may progress to the development of CL caused by *L*. *guyanensis*. Although the role played by neutrophils in *L*. *guyanensis*-infection is not clear, we hypothesize that this subset of individuals may attract an increased number of neutrophils at the site of infection which may be pathogenic to the host as demonstrated in animal models [48,49].

A drawback of our study is that we did not perform a delayed-type hypersensitivity test with *Leishmania* antigen in the control population to be sure they were all exposed to the vector harboring the parasite. Furthermore, our cytokine assay kit did not include IL-18.

In summary, we showed for the first time in leishmaniasis that the variant present in *CARD8* is associated with the development of CL in susceptible individuals infected with *L*. *guyanensis*. Interestingly, the SNV, associated with CL, correlated with circulating levels of plasma IL-8. Further studies are required to reproduce this association in another independent cohort of patients with CL and also in patients with Cl caused by other species of *Leishmania*.

## Supporting information

S1 Fig Correlation of circulating plasma level of IL-1β, IL-8 and TNFα with the SNV rs73944113 genotypes of *CARD8* in the codominant model. Analysis was performed by the Generalized Linear Model using the Anova one-way parametric model. P1 is the *p*-value adjusted for age and sex, while P2 is the *p*-value unadjusted. The bar represents the mean expressed in picogram/mL while the error bar is the standard error of the mean.
(TIF)

S2 Fig Correlation of circulating plasma level of IL-1β, IL-8 and TNFα with the SNV rs2043211 genotypes of *CARD8* in the codominant model. Analysis was performed by the Generalized Linear Model using the Anova one-way parametric model. P1 is the *p*-value adjusted for age and sex, while P2 is the *p*-value unadjusted. The bar represents the mean expressed in picogram/mL while the error bar is the standard error of the mean.
(TIF)

S3 Fig Correlation of circulating plasma level of IL-1β, IL-8 and TNFα with the SNV rs2288877 genotypes of *CARD8* in the codominant model. Analysis was performed by the Generalized Linear Model using the Anova one-way parametric model. P1 is the *p*-value adjusted for age and sex, while P2 is the *p*-value unadjusted. The bar represents the mean expressed in picogram/mL while the error bar is the standard error of the mean.
(TIF)

S4 Fig Correlation of circulating plasma level of IL-1β, IL-8 and TNFα with the SNV rs2288876 genotypes of *CARD8* in the codominant model. Analysis was performed by the Generalized Linear Model using the Anova one-way parametric model. P1 is the *p*-value adjusted for age and sex, while P2 is the *p*-value unadjusted. The bar represents the mean expressed in picogram/mL while the error bar is the standard error of the mean.
(TIF)

S5 Fig Correlation of circulating plasma IL-8 with the SNV rs2288877 A>G based on different inheritance models in patients with *Lg-*CL (cases), healthy controls (Controls), and the total individuals participating (patients with *Lg*-CL + controls). Analysis was performed by unpaired parametric T-tests between plasma levels of IL-8 and rs2288876 A>G genotypes P1 is the *p*-value adjusted for age and sex, while P2 is the *p*-value unadjusted. The bar represents the mean expressed in picogram/mL while the error bar is the standard error of the mean.
(TIF)

S6 Fig Correlation of circulating plasma IL-8 with the SNV rs2288876 A>G different inheritance models in patients with CL (cases), healthy controls (Controls), and the total individuals participating (patients with CL + controls). Analysis was performed by unpaired parametric T-tests between plasma levels of IL-8 and rs2288876 A>G genotypes. P1 is the *p*-value adjusted for age and sex, while P2 is the *p*-value unadjusted. The bar represents the mean expressed in picogram/mL while the error bar is the standard error of the mean.
(TIF)
