# Supplementary figures and images for: Variants of CARD8 in Leishmania guyanensis-cutaneous leishmaniasis and influence of the variants genotypes on circulating plasma cytokines IL-1β, TNFα and IL-8
Source: PLoS Negl Trop Dis. 2023 Jun 5;17(6):e0011416. doi: 10.1371/journal.pntd.0011416 (PMC10270566; doi:10.1371/journal.pntd.0011416)

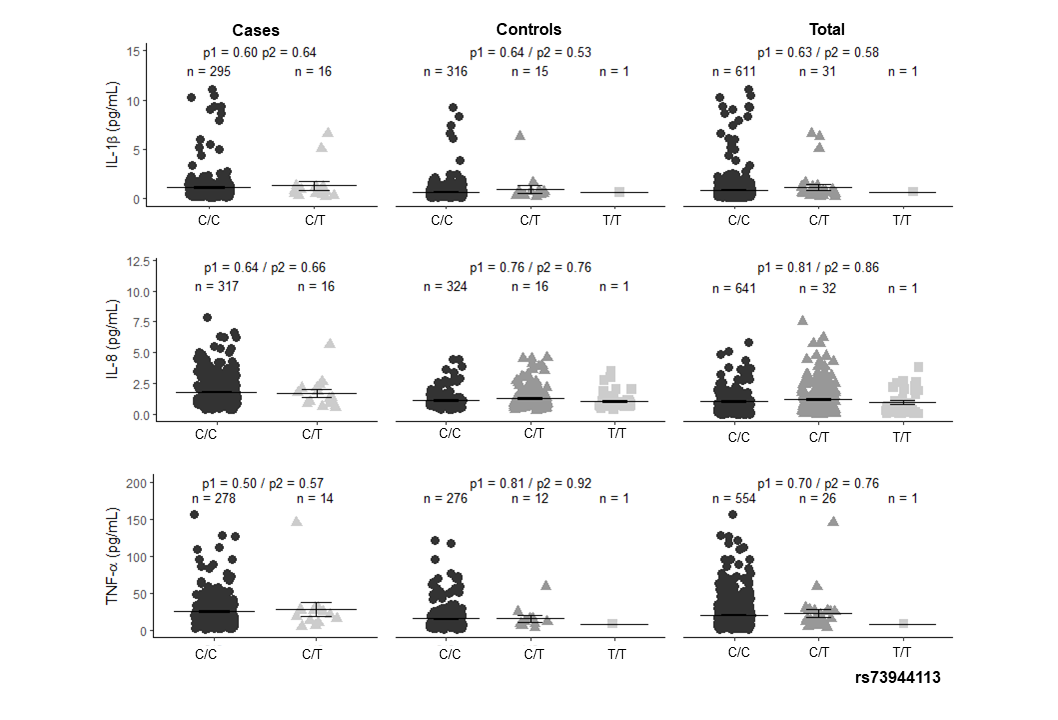

Supplement: S1 Fig — Analysis was performed by the Generalized Linear Model using the Anova one-way parametric model. P1 is the p-value adjusted for age and sex, while P2 is the p-value unadjusted. The bar represents the mean expressed in picogram/mL while the error bar is the standard error of the mean. (TIF) [file pntd.0011416.s001.tif]

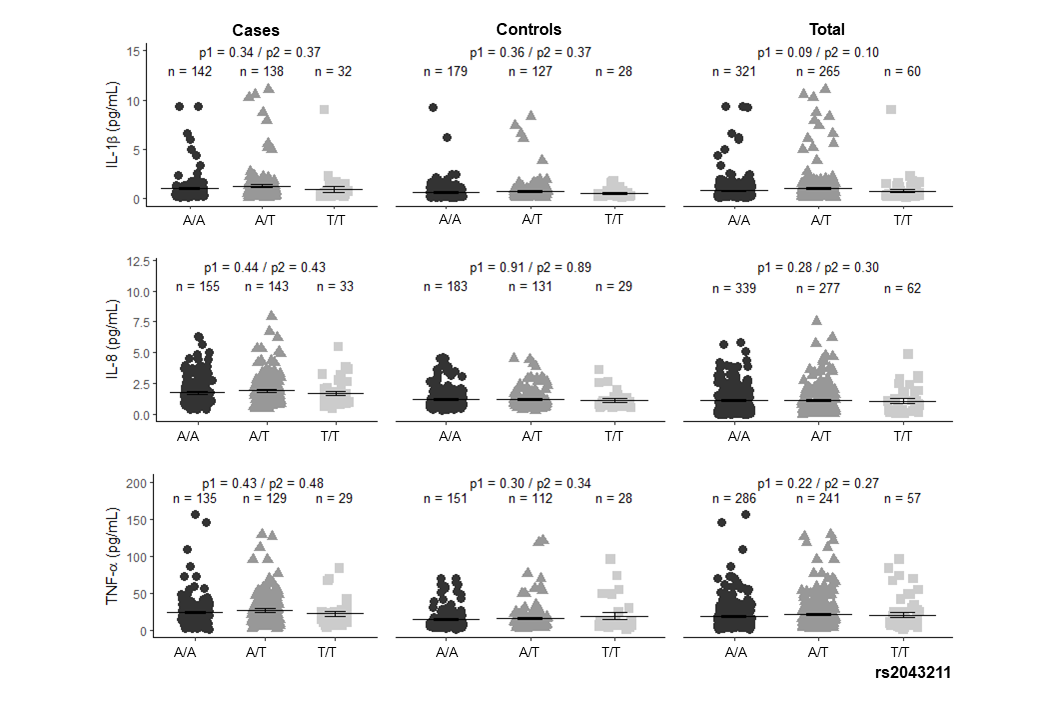

Supplement: S2 Fig — Analysis was performed by the Generalized Linear Model using the Anova one-way parametric model. P1 is the p-value adjusted for age and sex, while P2 is the p-value unadjusted. The bar represents the mean expressed in picogram/mL while the error bar is the standard error of the mean. (TIF) [file pntd.0011416.s002.tif]

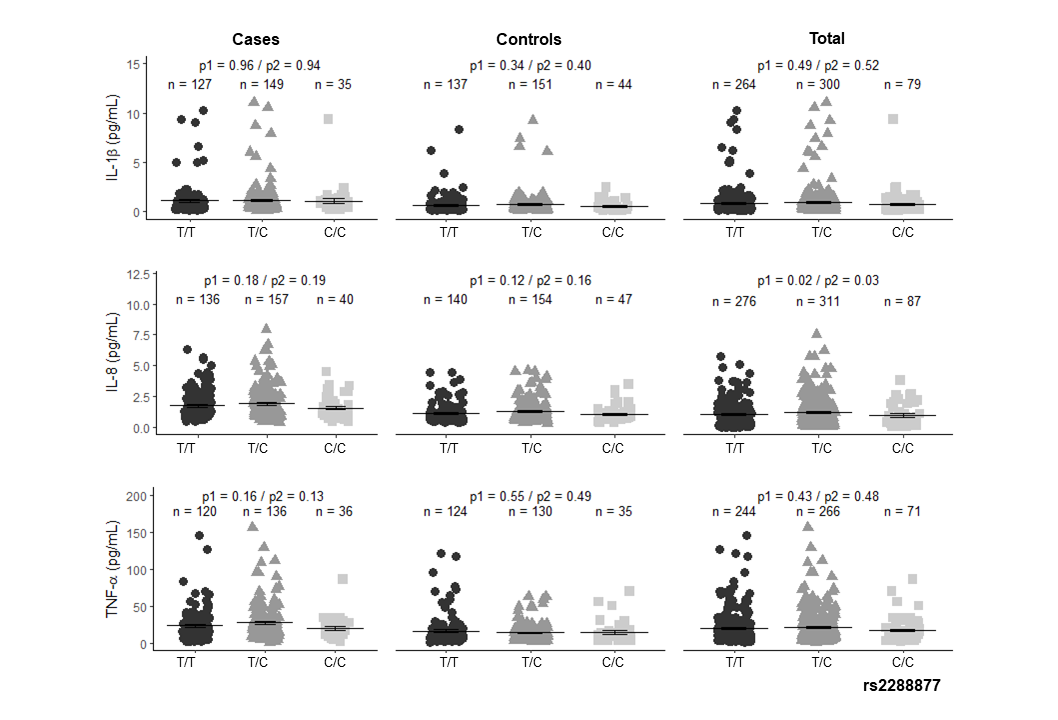

Supplement: S3 Fig — Analysis was performed by the Generalized Linear Model using the Anova one-way parametric model. P1 is the p-value adjusted for age and sex, while P2 is the p-value unadjusted. The bar represents the mean expressed in picogram/mL while the error bar is the standard error of the mean. (TIF) [file pntd.0011416.s003.tif]

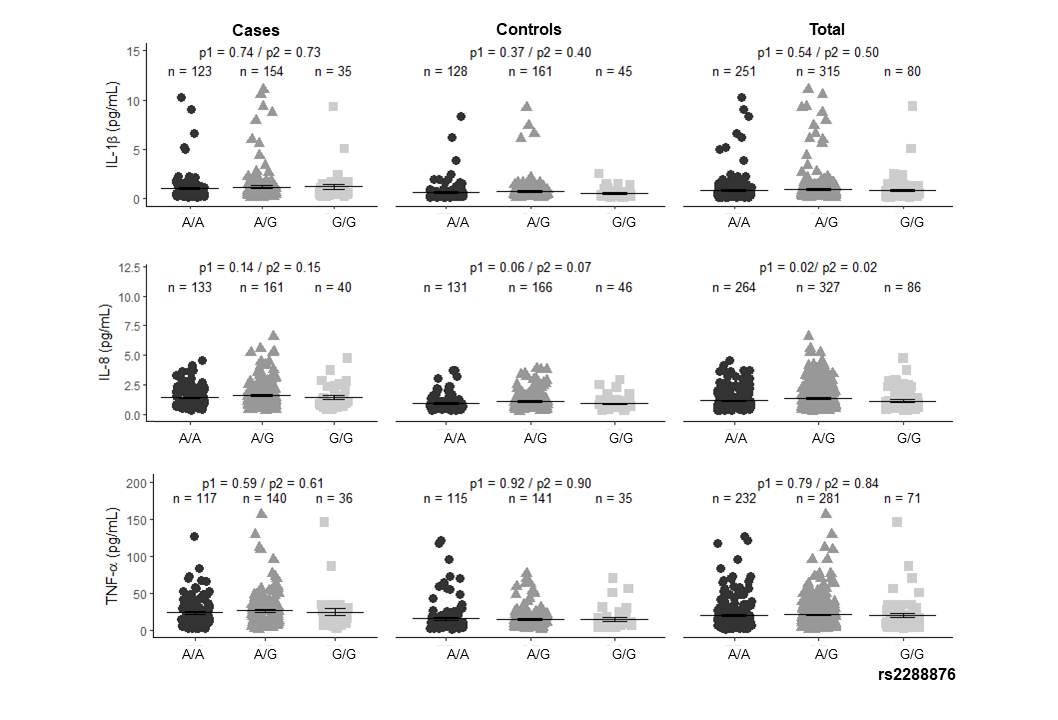

Supplement: S4 Fig — Analysis was performed by the Generalized Linear Model using the Anova one-way parametric model. P1 is the p-value adjusted for age and sex, while P2 is the p-value unadjusted. The bar represents the mean expressed in picogram/mL while the error bar is the standard error of the mean. (TIF) [file pntd.0011416.s004.tif]

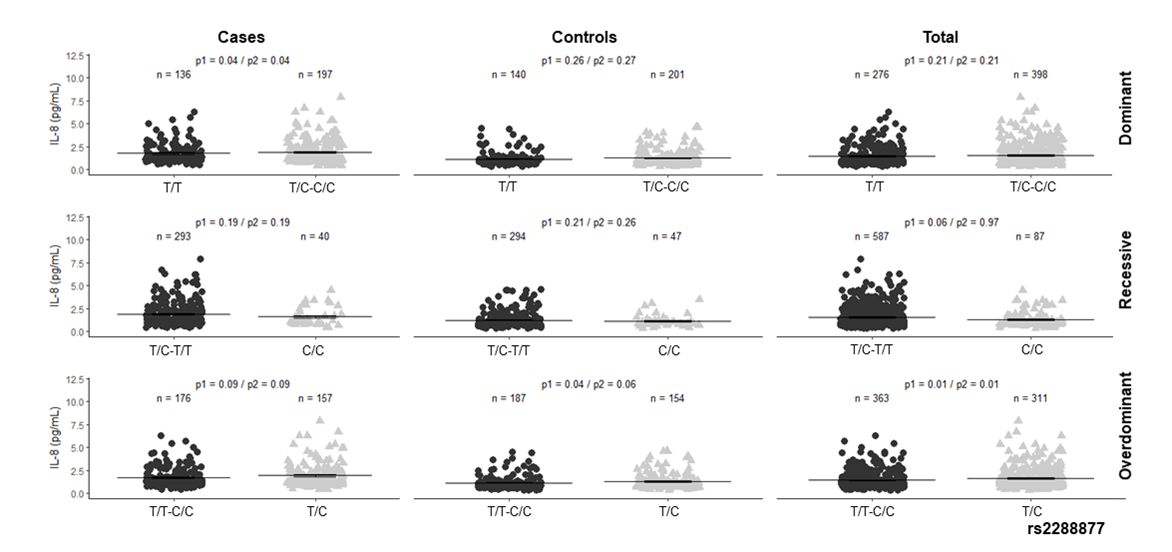

Supplement: S5 Fig — Analysis was performed by unpaired parametric T-tests between plasma levels of IL-8 and rs2288876 A>G genotypes P1 is the p-value adjusted for age and sex, while P2 is the p-value unadjusted. The bar represents the mean expressed in picogram/mL while the error bar is the standard error of the mean. (TIF) [file pntd.0011416.s005.tif]

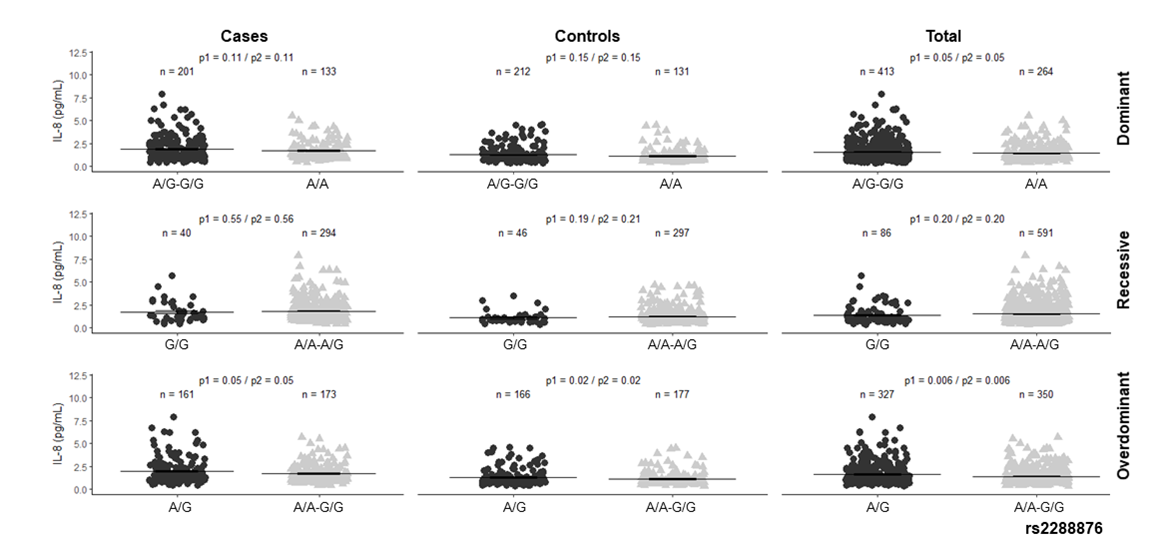

Supplement: S6 Fig — Analysis was performed by unpaired parametric T-tests between plasma levels of IL-8 and rs2288876 A>G genotypes. P1 is the p-value adjusted for age and sex, while P2 is the p-value unadjusted. The bar represents the mean expressed in picogram/mL while the error bar is the standard error of the mean. (TIF) [file pntd.0011416.s006.tif]
